# Matrix Metalloproteinase-9 Concentration in Peritoneal Fluid of Horses with and Without Strongyle Infection

**DOI:** 10.3390/ani16213352

**Published:** 2026-10-24

**Authors:** Kristina Kunze, Katharina Lisa Adamasky, Roswitha Merle, Georg von Samson-Himmelstjerna, Ina-Gabriele Richter, Heidrun Gehlen

**Affiliations:** 1 Equine Clinic, Freie Universität Berlin, 14163 Berlin, Germany; katharina.adamasky@gmx.de (K.L.A.); heidrun.gehlen@fu-berlin.de (H.G.); 2 Institute of Veterinary Epidemiology and Biostatistics, Freie Universität Berlin, 14163 Berlin, Germany; roswitha.merle@fu-berlin.de; 3 Institute for Parasitology and Tropical Veterinary Medicine, Freie Universität Berlin, 14163 Berlin, Germany; samson.georg@fu-berlin.de; 4 Department of Cell Biology, Research Centre of Medical Technology and Biotechnology, 99947 Bad Langensalza, Germany; irichter@fzmb.de

**Keywords:** cyathostomina, *Strongylus vulgaris*, inflammation, equine medicine, colic, gastrointestinal disease, parasitology

## Abstract

Strongyle infections are common in horses and can cause clinically relevant disease, yet their associated inflammatory responses remain incompletely understood. Matrix metalloproteinase-9 (MMP-9), a key mediator of extracellular matrix degradation and inflammation, has been proposed as a biomarker in various equine diseases. This study investigated whether strongyle infection is associated with increased MMP-9 concentrations in equine peritoneal fluid (PF). A total of 83 horses presenting with colic, weight loss, or faecal water were included, and strongyle infection was determined semi-quantitatively by combined sedimentation–flotation. MMP-9 concentrations in non-anticoagulated and citrate-anticoagulated PF were measured using ELISA, and non-parametric statistical tests were applied. MMP-9 measurements in non-anticoagulated samples were strongly correlated with the values from citrate-anticoagulated samples. No significant differences in MMP-9 concentrations, total nucleated cell count (TNCC), total protein (TP) or lactate were observed between strongyle-positive and -negative horses, nor across faecal egg count categories. In contrast, MMP-9 concentrations showed significant positive correlations with inflammatory parameters, including TNCC and TP, irrespective of strongyle infection. These findings indicate that peritoneal MMP-9 reflects non-specific inflammatory processes rather than strongyle infection. The lack of association may also reflect limitations of faecal egg counts and sampling site, suggesting that PF is not optimal for detecting parasite-specific inflammation.

## Simple Summary

Strongyle infections are widespread parasitic infections in horses and can contribute to clinical signs such as colic, weight loss, and abnormal faecal consistency. Understanding how these parasites influence inflammatory processes may improve diagnostic approaches and disease monitoring. This study investigated whether matrix metalloproteinase-9 (MMP-9), a protein involved in tissue remodelling and inflammation, could serve as a marker of strongyle-associated inflammation in the abdominal fluid of horses. Samples from 83 horses presenting with gastrointestinal complaints were analysed for evidence of strongyle infection and for MMP-9 concentrations. Of these, 23 horses tested positive for strongyle eggs. No differences in MMP-9 levels in peritoneal fluid were found between horses with and without strongyle infection, and MMP-9 concentrations were not associated with parasite egg counts in faeces. In contrast, MMP-9 levels were positively associated with established indicators of inflammation. These findings suggest that MMP-9 in abdominal fluid reflects non-specific inflammatory responses rather than inflammation specifically related to strongyle infection. The absence of a detectable association may also indicate limitations of faecal egg counts and abdominal fluid for assessing parasite-induced inflammation. This study advances our understanding of inflammatory biomarkers in horses and may support the development of targeted diagnostic tools for equine health management.

## 1. Introduction

Horses exposed to pasture are subjected to continuous exposure to gastrointestinal helminths, of which strongyles are the most prevalent and clinically significant group [1,2].

These parasites, including both cyathostomins (small strongyles) and large strongyles, have complex life cycles involving environmental and host-dependent stages and are endemic across equine populations in Europe [3,4]. Recent studies reported a high seroprevalence for *S. vulgaris* in the horse population in Berlin-Brandenburg (Germany), even though PCR assays revealed *S. vulgaris* and *S. edentatus* only in a small number of faecal samples [5]. In other European countries like Sweden there seems to be a high level of exposure to *Strongylus vulgaris*, as antibodies were shown to be present in serum from 62% of horses [6].

Although infections are often subclinical, both cyathostomins and large strongyles can cause significant disease. In particular, *S. vulgaris* is considered highly pathogenic due to larval migration through the arterial system, leading to vascular damage and thromboembolic colic, which may be fatal [7,8].

Cyathostomin larvae have the ability to enter hypobiosis for several months to up to three years; the following mass larval emergence can result in severe intestinal pathology and high mortality rates [9].

Contemporary parasite control strategies recommend selective anthelmintic treatment; however, standard coprological techniques do not enable species differentiation among strongyle eggs, thereby limiting identification of highly pathogenic species such as *S. vulgaris*. Consequently, there remains a significant risk that infections with this highly pathogenic species may go undetected and untreated [10,11]. Increasing anthelmintic resistance further highlights the need for improved diagnostic tools [12]. 

Matrix metalloproteinase-9 (MMP-9) is a zinc (II)-dependent multidomain enzyme that is responsible for the degradation of components of the extracellular matrix (ECM) [13]. The regulation of MMP-9 activity is highly complex and can be induced or depressed by multiple factors including cytokines, growth factors, hormones and cellular contact. The capacity of MMP-9 to degrade components of the extracellular matrix and to regulate the activity of various soluble proteins underpins its significant role in numerous physiological and pathological processes, including reproduction, growth, development, inflammation, and vascular and proliferative disorders [14]. In cases of infection with other nematoda like *Trichinella* spp., MMP-9 level in serum was a reliable inflammation marker in mice [15] and in humans [16]. Excessive MMP activity can cause ECM destruction, as seen in the lamellar basement membrane in laminitis and the articular cartilage in osteoarthritis in horses [17,18,19]. Increases in MMP activities have also been found in horses with recurrent airway obstruction, experimentally induced sepsis, kidney damage and ulcerative keratitis [20,21,22,23]. MMP-9 concentration in PF was found to be a biomarker with high diagnostic value in horses with sepsis and endotoxemia in equine colic [24]. Multiple studies have shown that measurement of MMP-9 activity was preferable in local samples [20,25,26,27,28,29,30]. 

Despite evidence from other species [15,16,31,32,33,34,35,36], the connection between parasitic infections and increased MMP-9 activity in horses has not yet been investigated. 

Due to the clinical importance of strongyle infections in horses and the potential utility of MMP-9 as a biomarker, the present study analysed PF from horses grouped according to the presence or absence of strongyle egg shedding. We hypothesised that strongyle infection is not associated with increased PF-MMP-9 concentrations.

## 2. Materials and Methods

### 2.1. Study Cohort

A total of 83 horses presented to the Equine Clinic of Freie Universität Berlin were included in this study. All horses exhibited clinical signs of colic of varying severity, chronic weight loss, or faecal water. The animals were not recruited specifically for the study; rather, they were presented to the clinic either spontaneously or as part of a planned visit for diagnostic evaluation and treatment between spring 2021 and autumn 2022.

### 2.2. Ethical Statement

All samples and parameters were obtained as part of the routine diagnostic workup performed at the hospital. All examinations were conducted within the scope of the individual case workup and primarily served diagnostic and therapeutic purposes.

Accordingly, the sampling procedures were not classified as animal experimentation. Written owner consent for the inclusion of their horses in the study was obtained during the admission process at the clinic.

### 2.3. Sample Collection

#### 2.3.1. Clinical Examination

Upon arrival at the clinic, all patients underwent a general clinical examination. In addition, parameters such as sex, breed, age, and reason for visiting were recorded. The standard clinical protocol at our clinic also includes an ultrasound of the abdomen following the standard FLASH-protocol [37], a rectal examination, and an examination using a nasogastric tube. Further diagnostics were arranged by the responsible veterinarian depending on the findings.

#### 2.3.2. Blood Samples

Blood samples were collected at admission via jugular venipuncture. Routine haematological analyses, including automated blood cell count, haematocrit, and total protein, were performed using EDTA-anticoagulated blood. Lactate and electrolyte concentrations were determined from Lithium-heparinized blood as the standard protocol of the hospital. The remaining blood was aliquoted into lithium heparin, citrate, and serum collection tubes. Samples were centrifuged for 10 min at 2260× *g* using a Hettich Rotofix 32 centrifuge (Andreas Hettich GmbH, Tuttlingen, Germany). Plasma and cell-free supernatant were pipetted and stored at −80 °C until further analysis.

#### 2.3.3. Peritoneal Fluid

Peritoneal fluid was collected as part of the clinical diagnostic work-up. Abdominocentesis was performed using a Tuohy epidural cannula, with or without local anaesthesia at the discretion of the attending veterinarian. Native PF was collected in serum collection tubes and subsequently aliquoted into EDTA, heparin, citrate, and plain (non-coated) tubes. Routine analyses, including TNCC, TP, and lactate, were performed immediately after sample collection. The remaining sample material was aliquoted and stored at −80 °C until further analysis. For the analysis, the results from the non-anticoagulated samples and those preserved in citrate medium were separated, and subgroups were created. A distinction was made between non-anticoagulated samples, citrate-anticoagulated samples, and participants with both types of samples.

#### 2.3.4. Faecal Samples

If horses were initially presented with colic, faecal samples for faecal egg count (FEC) were collected only after the first follow-up rectal examination had revealed no abnormalities. Samples were obtained either during rectal examination or collected from the stall immediately after first defecation following the unremarkable rectal examination. No anthelmintic drugs were administered during hospitalisation, before faecal sample collection. Samples were stored at +4 °C until combined flotation and sedimentation was performed. Therefore, at least 15 g of faecal material is thoroughly mixed with a minimum of 40 mL of tap water using a wooden spatula. The resulting suspension is poured through a tea strainer into a 250 mL beaker to remove coarse feed particles. The remaining liquid is pressed through the sieve into the beaker using the wooden spatula. The filtrate is then transferred into centrifuge tubes and centrifuged at 400× *g* for 10 min. After centrifugation, the supernatant is discarded, and the sediment is resuspended in concentrated sugar solution. This suspension is transferred into 15 mL centrifuge tubes, and 12 mL of concentrated sugar solution is added. The tubes are centrifuged at 200× *g* for 10 min. Following centrifugation, the entire surface of each tube is carefully transferred onto a microscope slide using a wire loop and covered with a cover slip. The sample is then examined under a microscope using either a 10× or 40× objective, and systematically scanned in a zigzag pattern [38].

Strongyle infection was defined based on the presence of strongyle eggs in faeces. The level of strongyle egg shedding was assessed semi-quantitatively and categorised as low (1–10 eggs), moderate (11–50 eggs), or high (>50 eggs). It is not possible to determine the number of eggs or oocysts per gram of faeces using this flotation technique.

### 2.4. Matrix Metalloproteinase 9

MMP-9 concentrations in citrated and non-anticoagulated PF were quantified using equine-specific ELISA kits (eqMMP-9 FAST ELISA Kit, fzmb GmbH, 99947 Bad Langensalza, Germany). The microtitre plates provided with the kit were pre-coated with an antibody specific for native equine MMP-9. Standards and samples were prepared in duplicate. Samples were diluted at ratios ranging from 1:10 to 1:100. After sample incubation, a horseradish peroxidase (HRP)-conjugated antibody specific for native equine matrix metalloproteinase-9 (MMP-9) was added and incubated. Subsequently, TMB substrate solution was added. Wells containing MMP-9, the enzyme-conjugated antibody, and the substrate developed a color reaction. The enzyme–substrate reaction was stopped by adding sulfuric acid solution, and absorbance was measured spectrophotometrically at 450 ± 10 nm using an ELISA microplate reader (Tecan, Männedorf, Switzerland). For the generation of the standard curve, the measured optical density values were plotted against the known concentration of each calibration standard. The curve was fitted using a 5-parameter logistic (5PL) model. MMP-9 concentrations in the samples were calculated from the measured optical density values using the resulting 5PL equation. The quantification range of the assay (the range between the lowest and highest detectable amounts) was 0.25–24 ng/mL per individual measurement. By analysing the sample at different dilution levels (up to maximum dilution of 1:100) the detectable concentration range can be extended.

Samples exceeding the upper limit of this range at a dilution of 1:100 were assigned a value of 2.400 ng/mL for statistical analysis (non-anticoagulated samples *n* = 4). Values below the lower limit of detection (<0.25 ng/mL) were assigned a value of 0.01 ng/mL for the purposes of statistical analysis (non-anticoagulated samples *n* = 12, citrate-anticoagulated samples *n* = 20). 

### 2.5. Statistical Analysis

The data were analysed and presented graphically using SPSS Statistics (Version 31.0.0.0 (117)). The data were tested for normal distribution using the Shapiro–Wilk test, Kolmogorov–Smirnov test, and visual inspection. The data were not normally distributed, and the logarithmic transformation did not achieve normality. Accordingly, non-parametric statistical tests were applied, including the Mann–Whitney U test and the Kruskal–Wallis test. The association between MMP-9 concentrations measured in non-anticoagulated PF and citrate-anticoagulated PF was assessed using Spearman’s rank correlation coefficient (ρ), as the distribution of the data was not assumed to be normal. Correlation coefficients were interpreted as a measure of the strength of the monotonic relationship between the two sampling media. As described above, the ELISA used for MMP-9 detection has a lower limit of detection of 0.25 ng/mL. Because correlation analyses require exact numerical values, calculations were performed both including and excluding values that had been technically assigned (i.e., values below the detection limit). Agreement between measurements obtained from non-anticoagulated and citrate-anticoagulated PF was further evaluated using Lin’s concordance correlation coefficient (CCC). In contrast to correlation analysis alone, the CCC assesses both precision and accuracy by quantifying the degree to which paired observations deviate from the line of perfect concordance (45° line).

In addition, Bland–Altman analysis was performed to assess the agreement between the two sampling media. For each pair of measurements, the difference between methods was plotted against their mean. The mean difference (bias) and the 95% limits of agreement, calculated as the mean difference ± 1.96 standard deviations of the differences, were determined to evaluate systematic bias and the range of disagreement between measurements.

Furthermore, associations between MMP-9 concentrations and other parameters measured in PF (TP, lactate and TNCC) were evaluated using Spearman’s rank correlation. For these analyses, values below the limit of detection (MMP-9 < 0.25 ng/mL) were excluded. Associations between TP, lactate, and TNCC concentrations in PF and parasitological findings were also investigated. As lactate and TNCC data were not normally distributed, the Mann–Whitney U test and Kruskal–Wallis test were performed. As TP data were normally distributed, an independent samples t-test and one-way analysis of variance (ANOVA) were applied.

## 3. Results

### 3.1. Study Population

A total of 83 horses were included in the study. The population included 36 different breeds, with German Sport Horse (13.3%) and Icelandic Horse (12.0%) being the most frequently represented breeds. The study population consisted of 26 mares, 4 stallions, and 53 geldings. The median age was 12 years, and the 25th and 75th percentiles were 7 and 18 years. 

Faecal examination revealed that 60 horses were negative for strongyle eggs, while 23 horses tested positive (11 horses showed a low-grade infection, 2 a moderate infection, and 10 a high-grade infection).

Non-anticoagulated PF samples were available from 78 horses. Of these, 57 horses were negative for strongyle eggs, whereas 21 horses were positive (11 low-grade, 1 moderate, and 9 high-grade infections). 

Citrated PF samples were obtained from 49 horses. Among these, 35 horses were negative, and 14 were positive for strongyle eggs (6 low-grade, 2 moderate, and 6 high-grade infections). 

Both non-anticoagulated and citrated PF samples were available for 44 horses. Within this subgroup, parasitological examination in 32 horses was negative and in 12 positive (6 low-grade, 1 moderate, and 5 high-grade infections). 

Assessment of data distribution demonstrated that several measured variables were not normally distributed. 

### 3.2. MMP-9 in Peritoneal Fluid

#### 3.2.1. Differences in Strongyle-Positive and Strongyle-Negative Horses

In non-anticoagulated PF, the median MMP-9 concentration was 31.67 ng/mL (25th–75th percentile: 13.45–73.76 ng/mL) in strongyle-negative horses. In strongyle-positive horses the median concentration was 31.86 ng/mL (25th–75th percentile: 13.44–111.95 ng/mL). 

In citrate-anticoagulated samples, the median MMP-9 concentration in strongyle-negative horses was 13.91 ng/mL (25th–75th percentile: 0.01–38.32 ng/mL), whereas in strongyle-positive horses it was 8.54 ng/mL (25th–75th percentile: 0.01–17.87 ng/mL).

Differences between horses with negative and FEC were analysed using the Mann–Whitney U test. No significant differences were detected for MMP-9 concentrations in non-anticoagulated (mean rank negative 39.21 vs. positive 40.29; *p* = 0.852; *n* = 78) or citrate-anticoagulated PF (mean rank negative 25.74 vs. positive 23.14; *p* = 0.551; *n* = 49). 

#### 3.2.2. Differences Between FEC Categories

Further analysis comparing graded FEC categories (negative, low-grade, moderate, and high-grade shedding) using the Kruskal–Wallis test also revealed no statistically significant differences for MMP-9 concentrations in non-anticoagulated PF (*p* = 0.878), MMP-9 concentrations in citrate-preserved PF (*p* = 0.615), or TNCC (*p* = 0.355). 

In non-anticoagulated samples, the median MMP-9 concentration in strongyle-negative horses was 31.67 ng/mL (25th–75th percentile: 13.45–73.76 ng/mL), whereas in low-grade positive horses the median MMP-9 concentration was 31.86 ng/mL (25th–75th percentile: 6.02–125.12 ng/mL); in moderate-grade positive horses the MMP-9 concentration was 80.68 ng/mL (*n* = 1) and in high-grade positive horses the median MMP-9 concentration was 17.59 ng/mL (25th–75th percentile: 13.44–111.95 ng/mL).

In citrate-anticoagulated samples, the median MMP-9 concentration in strongyle-negative horses was 13.91 ng/mL (25th–75th percentile: 0.01–38.32 ng/mL), whereas in low-grade positive horses the median MMP-9 concentration was 16.48 ng/mL (25th–75th percentile: 0.01–48.76 ng/mL); in moderate-grade positive horses the median MMP-9 concentration was 0.01 ng/mL (25th–75th percentile: 0.01–0.01 ng/mL) and in high-grade positive horses the median MMP-9 concentration was 8.54 ng/mL (25th–75th percentile: 0.01–13.52 ng/mL). 

### 3.3. Correlation and Agreement Between MMP-9 in Non-Anticoagulated and Citrate-Anticoagulated Samples

In 44 horses, paired PF samples were available: one non-anticoagulated sample and one sample collected in citrate. To determine whether citrate anticoagulation influenced measured MMP-9 concentrations, the correlation between MMP-9 concentrations in non-anticoagulated and citrate-anticoagulated PF was analysed. 

**Figure 1:**
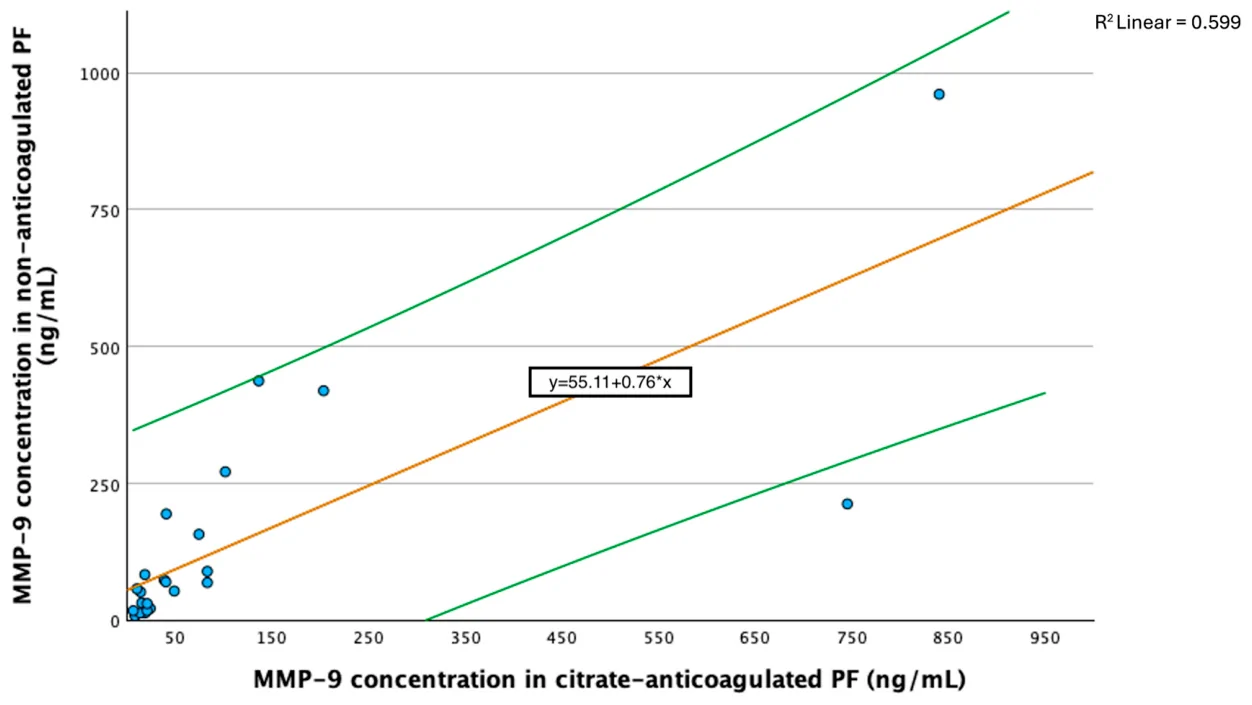
Relationship between MMP-9 concentrations measured in non-anticoagulated and citrate-anticoagulated peritoneal fluid. The central orange line represents the linear regression fit (y = 55.11 + 0.76x; *R*^2^ = 0.599), while the upper and lower green lines indicate the 95% prediction interval.

Spearman correlation analysis demonstrated a significant positive correlation between MMP-9 concentrations in non-anticoagulated and citrate-anticoagulated samples (ρ = 0.712, *p* < 0.001; *n* = 44) when all data were included. After excluding samples with concentrations below the detection limit of 0.25 ng/mL, the correlation was stronger (ρ = 0.856, *p* < 0.001; *n* = 26). The regression model explained 59.9% of the variance in MMP-9 concentrations in non-anticoagulated PF (*R*^2^ = 0.599). The regression equation was *y* = 55.11 + 0.76*x* (Figure 1), indicating that an increase of 1 ng/mL in MMP-9 concentration in citrate-anticoagulated PF was associated with an estimated increase of 0.76 ng/mL in non-anticoagulated PF. The regression coefficient was statistically significant (*B* = 0.764, SE = 0.130, *t* = 5.87, *p* < 0.001).

Lin’s CCC demonstrated a good agreement between the two media and was almost equivalent between all data (CCC = 0.848, *n* = 44) and after excluding samples with concentrations below the detection limit of 0.25 ng/mL (CCC = 0.836, *n* = 26). 

**Figure 2:**
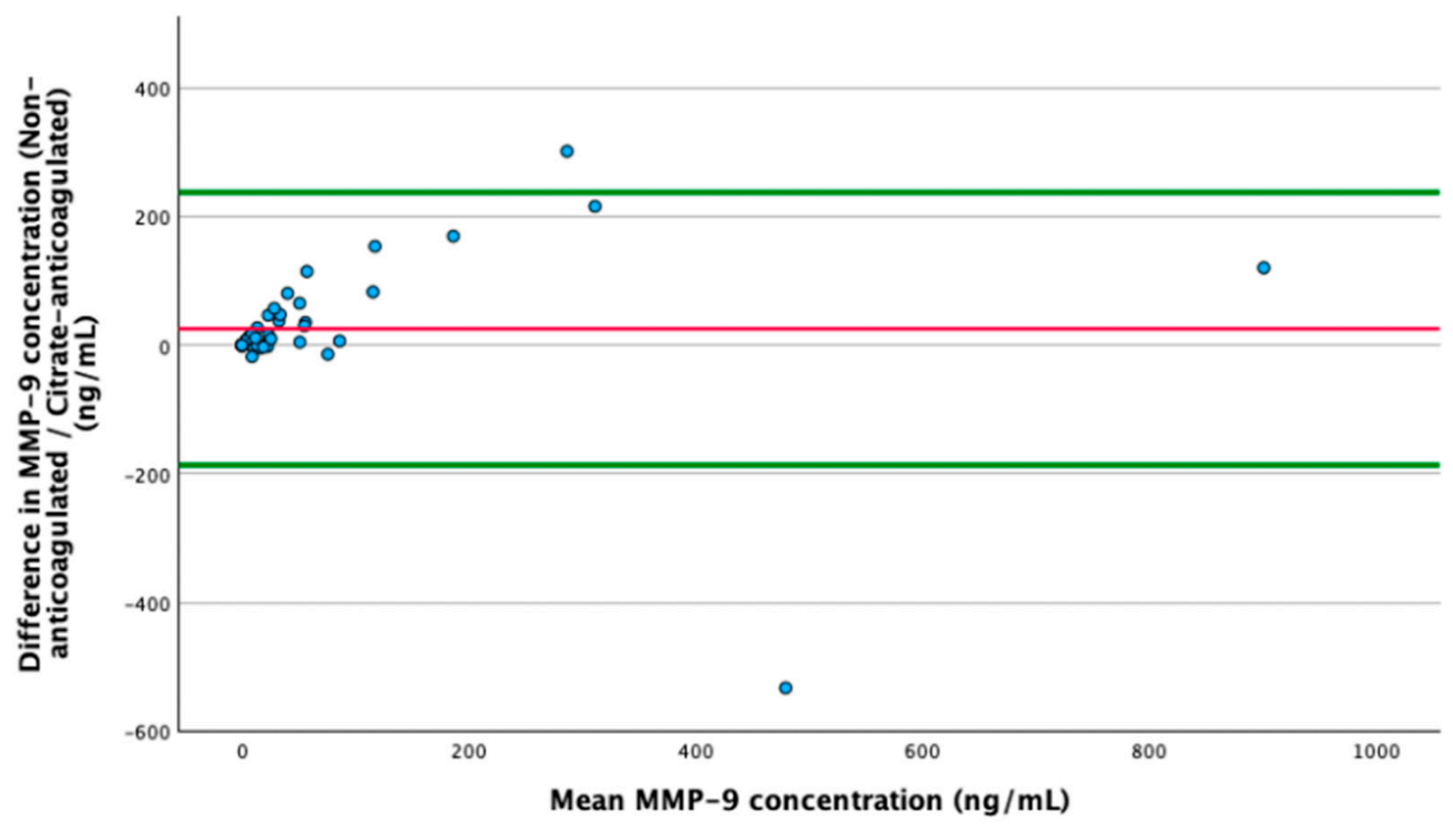
Bland–Altman plot showing agreement between MMP-9 concentrations measured in non-anticoagulated and citrate-anticoagulated peritoneal fluid. The central red line represents the mean difference (bias), while the upper and lower green lines indicate the 95% limits of agreement (mean difference ± 1.96 SD).

Bland–Altman analysis yielded a mean bias of 25.5 ng/mL, with 95% limits of agreement ranging from −186.8 to +237.7 ng/mL (Figure 2). No marked systematic bias was evident; however, variability between paired measurements increased at higher concentration ranges.

### 3.4. Total Nucleated Cell Count in Peritoneal Fluid

The median TNCC in PF was 0.86 × 10^9^/L (25th–75th percentile: 0.45–1.70 ×10^9^/L, *n* = 53) in strongyle-negative horses. In strongyle-positive horses, the median TNCC was 0.77 × 10^9^/L (25th–75th percentile: 0.54–1.50 × 10^9^/L, *n* = 19). Similarly, no significant difference was observed for TNCC (mean rank negative 36.00 vs. positive 37.89; *p* = 0.735; *n* = 72).

In low-grade positive horses, the median TNCC was 1.19 × 10^9^/L (25th–75th percentile: 0.77–1.50 × 10^9^/L); in moderate-grade positive horses, the median TNCC was 0.52 × 10^9^/L (25th–75th percentile: 0.49–0.54 × 10^9^/L); in high-grade positive horses, the median TNCC was 0.67 × 10^9^/L (25th–75th percentile: 0.40–1.36 × 10^9^/L). Further analysis comparing graded FEC categories (negative, low, moderate, and high) using the Kruskal–Wallis test also revealed no statistically significant differences for TNCC. 

### 3.5. Total Protein in Peritoneal Fluid

Comparison of total protein (TP) concentrations in PF between horses with negative and positive FEC results revealed no significant differences. In strongyle-positive horses, the mean TP concentration was 1.35g/dL with a standard deviation of 1.05 (*n* = 18). In strongyle-negative horses, the mean TP concentration was 1.43 g/dL with a standard deviation of 0.97 (*n* = 49). 

In low-grade positive horses, the mean TP concentration was 1.72 g/dL (standard deviation: 1.12, *n* = 9); in moderate-grade positive horses, the TP concentration was 0.20 g/dL (standard deviation: 0.00, *n* = 2); in high-grade positive horses, the mean TP concentration was 1.20 g/dL (standard deviation: 0.88, *n* = 7).

An independent samples *t*-test showed no effect of parasitological status on TP concentration (two-sided *p* = 0.941; Levene’s test *p* = 0.678). There were no statistically significant differences for TP concentration between different grades of FECs.

### 3.6. Lactate in Peritoneal Fluid

The median concentration of lactate in PF was 1.1 mmol/L (25th–75th percentile: 0.90–2.30 mmol/L, *n* = 53) in strongyle-negative horses. In strongyle-positive horses, the median concentration of lactate was 1.40 mmol/L (25th–75th percentile: 0.80–2.40 mmol/L, *n* = 20). Similarly, no significant difference was observed for the concentration of lactate in PF (mean rank negative 36.63 vs. positive 37.98; *p* = 0.809; *n* = 73).

In low-grade positive horses the median concentration of lactate in PF was 1.90 mmol/L (25th–75th percentile: 0.70–2.60 mmol/L, *n* = 10); in moderate-grade positive horses the median concentration of lactate was 0.65 mmol/L (25th–75th percentile: 0.40–0.90 mmol/L, *n* = 2); in high-grade positive horses the median lactate concentration was 1.40 mmol/L (25th–75th percentile: 1.20–2.35 mmol/L, *n* = 8). The Kruskal–Wallis test also revealed no statistically significant differences for lactate concentration between FEC categories. 

### 3.7. Correlation Between MMP-9 and TNCC, TP, and Lactate

Correlation analyses were also performed to evaluate associations between MMP-9 concentrations and selected PF parameters. 

In citrate-anticoagulated PF (*n* = 23), MMP-9 concentrations showed a strong positive correlation with TNCC (ρ = 0.645, *p* < 0.001) and with TP concentration (ρ = 0.675, *p* < 0.001). A moderate correlation was observed between MMP-9 concentration and lactate levels, although this was not significant (ρ = 0.303, *p* = 0.141).

In non-anticoagulated peritoneal fluid, MMP-9 concentrations were also strongly positively correlated with TNCC (ρ = 0.618, *p* < 0.001; *n* = 58). A moderate statistically significant correlation was identified between MMP-9 concentration and TP concentration (ρ = 0.325, *p* = 0.014; *n* = 57). A weak correlation was detected between MMP-9 concentration and lactate levels (ρ = 0.272, *p* = 0.035; *n* = 60). 

## 4. Discussion

The study investigated the relationship between the concentration of MMP-9 in PF and parasitological infestation with gastrointestinal strongyles in horses.

In the present study, a positive faecal flotation did not correlate with the concentration of MMP-9 in the peritoneal fluid. MMP-9 concentrations measured in non-anticoagulated and citrate-anticoagulated PF samples were not associated with the presence of strongyle eggs in the faeces, and FECs did not appear to influence MMP-9 concentrations. The significant positive correlation between values obtained from the two sample types suggests that citrate anticoagulation did not substantially affect the measured MMP-9 concentrations.

Combined sedimentation–flotation was used for the detection of faecal egg shedding, a method with high sensitivity for the detection of helminth eggs and shown to be sufficient and more sensitive than Mini-FLOTAC and FECPAK^G2 [39]. 

The absolute number of strongyle eggs shed in equine faeces has been shown to be inhomogeneously distributed; consequently, faecal egg counts may vary in the absolute number of eggs detected within a single sample or between samples from the same horse on consecutive days [40]. Faecal egg counts determined using the Cornell–McMaster technique in samples collected over four consecutive days showed greater day-to-day variation than would be expected under consistent egg shedding [41]. 

There remains a misconception that the number of detected eggs reflects parasite burden; however, FEC does not necessarily correspond to worm load and therefore provides limited information regarding parasitic involvement in disease [42]. 

In a large retrospective study, it was demonstrated that a direct linear correlation between parasite burden and FEC is not present. However, horses with strongyle FECs below the cut-off of 500 eggs per gram of faeces exhibited a lower worm burden [43]. In addition, egg shedding is known to vary considerably both within and between individuals, and phenomena such as hypobiosis—particularly in cyathostomins—can result in the absence of egg shedding despite active infection. Furthermore, FEC primarily represents luminal adult stages and does not capture larval stages migrating within host tissues [40,41,42]. Although the McMaster technique would have enabled a more standardized quantitative assessment of faecal egg shedding, combined sedimentation–flotation was selected for its high sensitivity in detecting helminth eggs. Nevertheless, the lack of standardized quantitative faecal egg counts should be considered a limitation of the present study, as potential associations between the intensity of egg shedding and MMP-9 concentrations could not be fully assessed. 

In cyathostominae development from third-stage larvae to fourth-stage larvae takes place within the intestinal wall of ceacum and colon. However, larvae of large strongyles undergo extensive migration through the host’s body, including the blood vessels, which may sustain considerable damage as a result. The larvae subsequently return to the lumen of the gastrointestinal tract, where they mature and begin egg shedding [3]. A major limitation of conventional coprological examination is that strongyle eggs are morphologically indistinguishable at the genus and species level. Thus, a positive strongyle egg count alone provides no information on whether cyathostomines or large strongyles are present and, importantly, does not allow the specific detection of clinically meaningful species such as *S. vulgaris* [10,11]. Therefore, the lack of detectable association between FEC and MMP-9 concentrations may reflect methodological limitations rather than a true absence of an underlying biological relationship. 

In addition, PF may not be the optimal biological matrix for detecting MMP-9 changes associated with strongyle infection or for elucidating the underlying pathomechanisms. PF is anatomically closely related to the abdominal organs, and numerous diseases of the intestinal wall can alter its composition [44,45]. Abdominocentesis is frequently performed as part of the diagnostic workup of various pathologies of the gastrointestinal tract or abdominal cavity. Horses included in the present study have been presented with clinical signs of colic, weight loss, or faecal water, which have been reported in association with strongyle infection. The obtained PF can provide information regarding inflammatory, ischaemic, infectious, or neoplastic diseases within the abdominal cavity [46,47,48]. Previous studies have reported differences in MMP-9 concentrations according to the underlying cause of colic [20,24]. Therefore, other gastrointestinal pathological processes responsible for clinical signs may have a stronger influence on MMP-9 concentration in PF than the strongyle infection itself and may mask mild changes caused by parasite-induced inflammation. The absence of a healthy control group represents an additional limitation of the present study. As peritoneal fluid samples were obtained from clinical patients undergoing diagnostic abdominocentesis potential confounding effects of underlying non-parasitic gastrointestinal diseases cannot be excluded. Future prospective studies involving healthy control and horses with confirmed strongyle infections, ideally with longitudinal sampling may help to distinguish parasite-associated changes in MMP-9 concentrations from those caused by other gastrointestinal pathologies.

It is conceivable that measurement of MMP-9 within the intestinal wall—where larval migration occurs—would reveal differences between infected and non-infected horses. In the present study, abdominocentesis was selected because it is considerably less invasive than a full-thickness biopsy of the intestinal wall. In studies involving *Toxocara canis*, enzyme activity was measured directly in affected tissues such as lung, muscle, and brain during larval migration [35], where a significant increase in MMP-9 was observed during the first week post-infection. In mice, infective third-stage larvae (L3) of *Angiostrongylus cantonensis* migrate via the bloodstream to the brain after ingestion; infected animals exhibited significantly elevated MMP-9 activity in the brain compared with uninfected controls [33]. Although peritoneal fluid may reflect inflammatory changes within the abdominal cavity, analysis of intestinal wall tissue may provide more direct insight into parasite-associated intestinal inflammation. Additionally, potential dilution effects in PF could further mask measurable differences. Consequently, the use of PF may limit the ability to detect parasite-associated changes in MMP-9 concentrations.

Conversely, there is evidence that parasitic infection may modulate the immune response and reduce MMP-9 concentrations [31]. In rats treated with excretory–secretory products of *Trichinella spiralis* muscle larvae, milder clinical signs were observed following induction of experimental autoimmune encephalomyelitis (an animal model of multiple sclerosis in humans). The authors suggested a regulatory mechanism involving IL-10-mediated modulation of MMP-9 expression triggered by *T. spiralis* excretory–secretory products [31]. These findings suggest that parasite-derived products may have immunomodulatory effects and potentially attenuate inflammatory responses. However, the immune response to gastrointestinal parasitic infection remains incompletely understood, and its effect may vary depending on parasite species, infection burden and host immune status.

The relatively small number of strongyle-positive horses, particularly within the moderate-grade group represents a limitation in terms of statistical power. The uneven distribution of level of strongyle egg shedding, combined with high data variability and the presence of outliers, may have reduced the ability to detect significant differences. Additionally, the non-normal distribution of the data further complicates statistical analysis. As a result, the possibility of a type II error (failure to detect an existing effect) must be taken into account and further studies including a larger number of strongyle-positive animals should be carried out.

A recent study demonstrated that horses developing Systemic Inflammatory Response Syndrome (SIRS) during hospitalisation exhibited significantly higher peritoneal fluid TNCC, TP, and lactate concentrations compared with horses that did not develop this condition [49]. Previous work by Barton and colleagues likewise reported that SIRS is associated with increased concentrations of MMP-9 [24]. The findings of the present study are consistent with these observations, as MMP-9 concentrations in PF were associated with inflammatory parameters such as TNCC, TP and lactate irrespective of the parasitological status of the horses.

Furthermore, MMP-9 concentrations have been shown to correlate positively with neutrophil percentages, TP, and TNCC [46]. These authors suggested that MMP-9 primarily reflects neutrophil-driven inflammatory processes. The observed positive correlations in this study between MMP-9 concentrations and inflammatory parameters such as TNCC and TP suggest that MMP-9 primarily reflects non-specific inflammatory processes. Acute inflammatory conditions associated with gastrointestinal disease, including ischemia or endotoxemia, may have masked any subtle effects attributable to parasitic infection. 

The present study demonstrates a significant positive correlation between MMP-9 concentrations measured in non-anticoagulated and citrate-anticoagulated peritoneal fluid. However, correlation alone is not sufficient to draw a conclusion about the effect of citrate-anticoagulation on assay performance.

In addition to correlation analysis, agreement between the two sample types was assessed using Lin’s CCC and Bland–Altman analysis. The CCC indicated good concordance between non-anticoagulated and citrate-anticoagulated samples (CCC = 0.84), while Bland–Altman analysis revealed a relatively small mean bias of 25.5 ng/mL. Together, these findings suggest that citrate anticoagulation may have only limited effect on measured MMP-9 concentrations in terms of systemic bias. 

The observed improvement in correlation after exclusion of values below the detection limit highlights an important methodological aspect. Due to the technical constraints of the ELISA, values below 0.25 ng/mL had to be assigned an arbitrary low value (0.01 ng/mL) for statistical purposes. While this approach enables the inclusion of all samples in the analysis, it introduces artificial uniformity and may obscure true biological variability, thereby weakening correlation estimates. The stronger correlation observed after excluding these values likely reflects a more accurate representation of the relationship between the two sample types, as it is based solely on reliably quantified concentrations within the assay’s validated range.

From a practical perspective, confirming that citrate anticoagulation does not substantially affect MMP-9 measurements could support the use of citrate-anticoagulated PF for MMP-9 analysis in horses. Citrate anticoagulation may be advantageous in clinical and research settings, because it helps prevent clot formation by chelating calcium, thereby facilitating sample handling and processing. Although the combination of strong correlation, good concordance, and minimal systematic bias indicates agreement between the two sample types, potential effects of citrate anticoagulation on antibody binding or ELISA performance cannot be excluded without further analytical validation. 

Nevertheless, the Bland–Altman analysis demonstrated relatively wide limits of agreement (−186.8 to +237.7 ng/mL), indicating that substantial differences may occur between paired measurements in individual samples. Consequently, although MMP-9 concentrations in non-anticoagulated and citrate-anticoagulated peritoneal fluid samples appear comparable at the group level, the two sample types should not be considered fully interchangeable for individual MMP-9 measurements. 

The reduction in sample size after exclusion of low-concentration values (from 44 to 26 paired samples) should also be considered when interpreting the results. This may indicate that a substantial proportion of samples had MMP-9 concentrations near or below the detection limit, which could be relevant in horses with low-grade or absent peritoneal inflammation. In such cases, methodological limitations of the assay may affect sensitivity and the ability to detect subtle differences between non-anticoagulated and citrate-anticoagulated samples. To draw a valid conclusion, a study with a larger sample size would need to be conducted.

Overall, the findings indicate that non-anticoagulated and citrate-anticoagulated peritoneal fluid provide comparable measurements of MMP-9 within the quantifiable range of the assay. Nevertheless, the wide limits of agreement and increasing variability at higher concentration warrant cautious interpretation and the potential effect of citrate on assay performance should be assessed independently. Future studies could further investigate the agreement between methods to evaluate the stability of MMP-9 under different storage conditions to better define optimal sample handling protocols for clinical practice. 

The study population reflects the real clinical patient population in this region. 

A prospective study including selected healthy horses examined before and after an experimental strongyle challenge might provide clearer information.

## 5. Conclusions

The present study found no association between FECs of equine strongyles and MMP-9 concentrations, TNCC, TP, or lactate in equine peritoneal fluid, regardless of sample preservation method. The absence of a relationship between strongyle egg shedding and peritoneal MMP-9 concentrations suggests that FECs may not adequately reflect parasite burden and therefore parasitic-induced inflammatory processes within the abdominal cavity. Instead, MMP-9 levels were positively correlated with established inflammatory markers, indicating that it primarily reflects neutrophil-driven inflammation rather than egg shedding intensity of strongyle endoparasites. These results highlight important limitations of using FECs as a surrogate for parasite-associated pathology and suggest that peritoneal fluid may not represent the optimal compartment for detecting parasite-specific inflammatory responses. 

Future studies should therefore focus on controlled experimental designs and, where feasible, include direct assessment of intestinal tissues to better elucidate the role of MMP-9 in parasite–host interactions. A deeper understanding of these mechanisms may help to clarify the complex and potentially bidirectional immunomodulatory effects of gastrointestinal parasites in horses.

## Data Availability

The data underlying the results of this study are available from the authors upon reasonable request.

## Abbreviations

The following abbreviations are used in this manuscript:

DSS	Dextran sodium sulfate
CCC	Lin’s Concordance Correlation Coefficient
ECM	extracellular matrix
ELISA	enzyme-linked immunosorbent assay
FEC	faecal egg count
L3	third-stage larvae
L4	fourth-stage larvae
MMP	Matrix Metalloproteinase
PCR	polymerase chain reaction
PF	peritoneal fluid
SIRS	Systemic Inflammatory Response Syndrome
TNCC	total nucleated cell count
TP	total protein
